# Class III Phosphatidylinositol-3 Kinase/Vacuolar Protein Sorting 34 in Cardiovascular Health and Disease

**DOI:** 10.1007/s12265-024-10581-z

**Published:** 2025-01-16

**Authors:** Yuanjun Shen, Jason P. Gleghorn

**Affiliations:** 1 Departments of Biomedical Engineering, University of Delaware, Newark, DE USA; 2 Biological Sciences, University of Delaware, Newark, DE USA; 3 School of Pharmacy and Pharmceutical Sciences, Binghamton University, Johnson City, NY USA

**Keywords:** Cardiovascular health, Cardiovascular disease, Vacuolar protein sorting 34

## Abstract

Phosphatidylinositol-3 kinases (PI3Ks) play a critical role in maintaining cardiovascular health and the development of cardiovascular diseases (CVDs). Specifically, vacuolar Protein Sorting 34 (VPS34) or PIK3C3, the only member of Class III PI3K, plays an important role in CVD progression. The main function of VPS34 is inducing the production of phosphatidylinositol 3-phosphate, which, together with other essential structural and regulatory proteins in forming VPS34 complexes, further regulates the mammalian target of rapamycin activation, autophagy, and endocytosis. VPS34 is found to have crucial functions in the cardiovascular system, including dictating the proliferation and survival of vascular smooth muscle cells and cardiomyocytes and the formation of thrombosis. This review aims to summarize our current knowledge and recent advances in understanding the function and regulation of VPS34 in cardiovascular health and disease. We also discuss the current development of VPS34 inhibitors and their potential to treat CVDs.

**Graphical Abstract:**

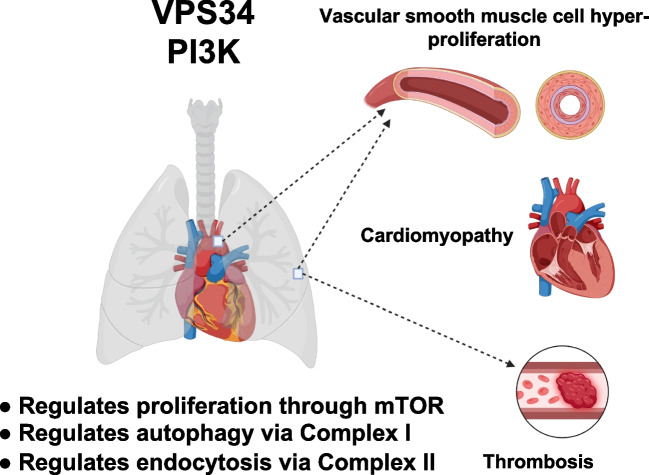

## Introduction

Cardiovascular diseases (CVDs) are the leading causes of global mortality [1, 2]. As an umbrella term, CVDs encompass various disorders affecting the heart and blood vessels, including ischemic heart disease, coronary artery disease, cerebrovascular disease, hypertension, peripheral vascular disease, congenital heart disease, rheumatic heart disease, cardiomyopathies, and cardiac arrhythmias [3]. Extensive research on several cell signaling pathways has provided a better understanding of CVD progression, aiding in the development of effective therapies. For instance, pulmonary vascular remodeling in pulmonary arterial hypertension (PAH) is tied to dysregulated Wnt/β-catenin [4], HIPPO [5, 6], PI3K/Akt/mTOR [7–9], and activin signaling [10] pathways. Knowledge of these pathways has, in part, contributed to advancements in clinical therapies, exemplified by the recent FDA approval of sotatercept, an activin signaling inhibitor, for the treatment of PAH [11].

Among the various signaling pathways contributing to maintaining cardiovascular health or causing CVDs, PI3K/Akt/mTOR pathway plays a pivotal role. PI3K, a family of lipid-modifying kinases, primarily phosphorylates phosphatidylinositol (PtdIns) and phosphoinositides, which are involved in processes such as signal transduction, endocytosis, vesicle trafficking, cortical remodeling, amino acid sensing, autophagy, endosomal signaling, cytokinesis, secretion, and Akt/mTOR activation [12, 13]. Notably, research has shown that upregulated canonical PI3K/Akt/mTOR signaling drives the proliferation of pulmonary arterial vascular smooth muscle cells (PAVSMCs) in PAH [14], while insulin deficiency-induced Class I PI3K signaling defects in the heart are associated with contractile dysfunction [15]. Current FDA-approved PI3K/Akt/mTOR inhibitors are limited to cancer therapy [16], largely due to their off-target effects and toxicities [17, 18]. However, new drug delivery methods, such as mTOR inhibitor-coated drug-eluting stents, are being tested in cardiovascular clinical trials [19]. At the same time, increasing research is also focusing on targeting other components of the PI3K/Akt/mTOR pathway, leading to growing interest in vacuolar protein sorting 34 (VPS34), also known as PIK3C3—the sole member of Class III PI3K. Our research, along with others, has demonstrated that VPS34 regulates PAVSMC proliferation [20, 21], cardiac hypertrophy and heart failure [22–24], and thrombosis [25, 26].

In this review, we will summarize the structure and regulation of VPS34, as well as current findings on its role in vascular smooth muscle cell proliferation and its functions in cardiovascular health and disease.

## VPS34 Structure, Regulation, and Function

###  Structures of PI3Ks

**Fig. 1:**
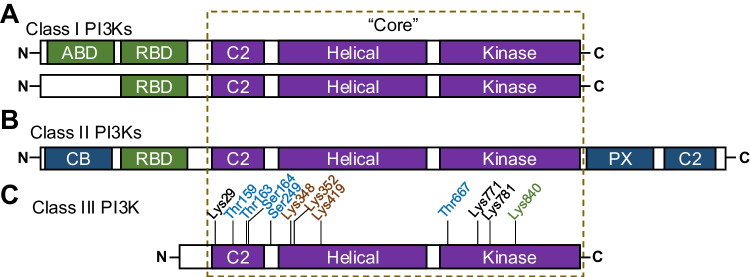
Structure of the three PI3K classes.** A** The schematic representation of the sequence of Class I PI3K enzymes. ABD – Adaptor-binding domain; RBD – Ras-binding domain. **B** The sequence of Class II PI3K enzymes. CB – Clathrin-binding domain; PX – Phox-homology domain. **C** The sequence of VPS34/Class III PI3K enzyme. Residue colors represent the posttranslational modifications (PTM). Blue residues: Phosphorylation; Black residues: Acetylation; Orange residues: Ubiquitination; Green residues: SUMOylation

PI3Ks are evolutionarily conserved and are organized into Class I, II, or III [27–29]. All three classes of PI3Ks contain the PI3K “core” domains: C2 domain, Helical domain, and Kinase domain. Class I PI3Ks also have a Ras-binding domain and can have an Adaptor-binding domain (Fig. 1A). Class II PI3Ks have a Ras-binding domain, a clathrin-binding domain, a phox-homology domain, and a C2 domain (Fig. 1B). Class III PI3K, with one isoform, only has the “core” domains **(**Fig. 1C) [12, 30].

**Fig. 2:**
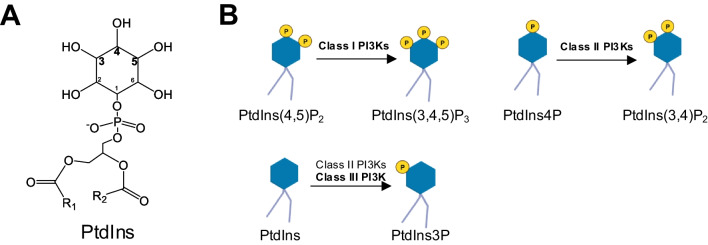
Details of PI3K-mediated PtdIns phosphorylation. **A** The structure of Phosphatidylinositol (PtdIns). R1 represents the carbon chain in stearic acid, and R2 represents the carbon chain in arachidonic acid. **B** The reactions catalyzed by PI3Ks

**Fig. 3:**
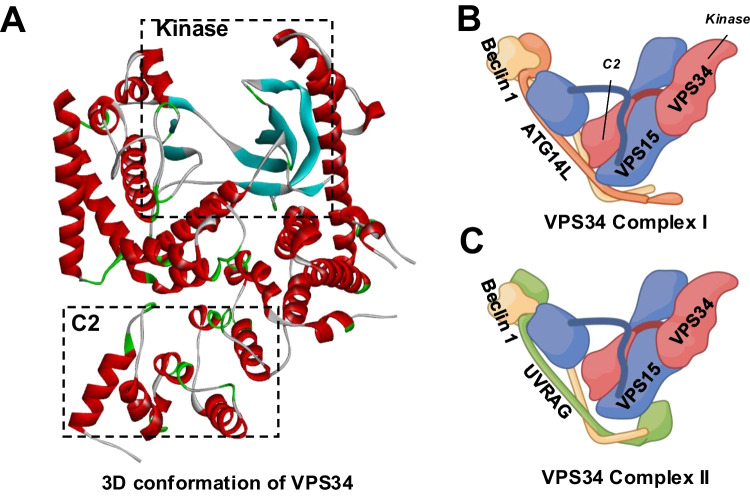
The structures of VPS34 and VPS34 complexes. **A** The three-dimensional structure of VPS34 is adapted from *4OYS* (rcsb.org). Boxes with dotted lines represent the locations of the C2 and the kinase domains. The water and the inhibitor molecules were removed, and the structure was visualized by Discovery Studio Visualizer (BIOVIA, Dassault Systèmes, v21.1.0.20298). **B** VPS34 Complex I. **C** VPS34 Complex II. The descriptive structures of VPS34 Complexes (Created with BioRender.com). Red: VPS34; Blue: VPS15; Yellow: Beclin 1; Orange: ATG14L; Green: UVRAG

The PI3K class dictates the phosphoinositide (PtdIns) substrate(s) and the subsequent phosphorylation sites, leading to different functions of the PI3Ks (Fig. 2A, B) [12]. Vacuolar Protein Sorting 34 (VPS34), also known as PIK3C3, is the only Class III PI3K family member. Compared to the Class II PI3Ks, VPS34 is a more efficient enzyme to produce PtdIns 3-phosphate (PtdIns3P)(Fig. 2B), which regulates vesicle trafficking and lysosomal protein transport [13]. The 3D structure of VPS34 is described as “a completely ordered phosphoinositide-binding loop”, which is critical for the binding of PtdIns [31, 32], the only substrate of the mammalian VPS34 [23] (Fig. 3A). When folded over the helical domain, the C-terminal catalytical kinase domain phosphorylates PtdIns into PtdIns3P. The truncation of 10 residues from the C-terminus significantly disrupts VPS34 catalytical activity [31]. The improved understanding of the VPS34 structure has resulted in new designs of selective VPS34 inhibitors [33], as discussed below.

### Regulation of VPS34

VPS34 activity is regulated by posttranslational modifications (PTMs) (phosphorylation, acetylation, ubiquitination, and SUMOylation) and complex formation with other structural and regulatory proteins [34–36]. Activated VPS34 further activates the mTOR pathway by suppressing the key mTOR Complex 1 (mTORC1) negative regulator, tuberous sclerosis complex 2 (TSC2) [9, 37, 38], and promotes autophagy and endocytosis.

#### VPS34 Activity Regulation by Phosphorylation

VPS34 phosphorylation is one of the most studied PTMs in VPS34 regulation. Research has revealed many VPS34 phosphorylation residues, including Thr159, Thr163, Ser164, Ser249, and Thr677. Most reported VPS34 phosphorylation modifications compromised the activation of VPS34, such as the phosphorylation mediated by cyclin-dependent kinase 1 (CDK1) and CDK5 on Thr159 [39], or by 5’AMP-activated protein kinase (AMPK) on Thr163 [40, 41]. However, protein kinase D-mediated phosphorylation on Thr677 activates VPS34 [42] (Fig. 1C, residues in blue, Table 1). Reports also showed that phosphorylation of VPS34 on Ser164 and Ser249 reduced VPS34 activity [41], the regulator(s) of which are unknown. The impact of phosphorylation on these sites of VPS34 on cardiovascular health and diseases remains to be further elucidated.

**Table 1:** VPS34 posttranslational modifications

Posttranslational modification	Residue	Regulation	Function	References
Phosphorylation	Thr159	Phosphorylated by CDK1 and CDK5	Inactivation	[39]
	Thr163	Phosphorylated by AMPK		[40, 41]
	Ser164	Regulator(s) not clear		[41]
	Ser249			
	Thr667	Phosphorylated by PKD	Activation	[42]
Acetylation	Lys29	Acetylated by p300	Inactivation	[43]
	Lys771			
	Lys781			
Ubiquitination	Lys348	K63-linked poly-ubiquitination mediated by the UBC13/UEV-1/CHN-1 complex	Activation	[44]
	Lys352			
	Lys419	K48-linked poly-ubiquitination mediated by NEDD4	Inactivation	[45]
SUMOylation	Lys840	SUMOylated by KRAB-associated protein 1	Activation	[21, 46]

#### VPS34 Deactivation by Acetylation

VPS34 is acetylated by p300 on Lys29 of the C2 domain, and on Lys771 and Lys781 of the catalytical domain (Fig. 1C, residues in black, Table 1). Lys29 acetylation compromised the association between VPS34 and Beclin 1, while Lys771 and Lys781 acetylation decreased VPS34 activity by blocking the PtdIns binding site. Su et al. found that the deacetylation of the above residues is essential for VPS34 activation [43]. Whether VPS34 acetylation plays a role in CVD progression remain further investigation.

#### VPS34 Stability Regulation by Ubiquitination

VPS34 ubiquitination is regulated by the UBC13/UEV-1/CHN-1 complex, which mediates K63-linked polyubiquitination at Lys348 and Lys352, and by NEDD4 (neural precursor cell expressed developmentally down-regulated protein 4), which mediates K48-linked polyubiquitination at Lys419 (Fig. 1C, residues in orange, Table 1). However, these two forms of ubiquitination have differing effects on VPS34 stability. Mutations that impair K63-linked polyubiquitination not only lead to increased VPS34 degradation but also downregulate VPS15 [44], a key component of VPS34 complexes. In contrast, NEDD4 inhibits K48-linked polyubiquitination, which stabilizes VPS34 and promotes downstream autophagy [45]. While direct research on the role of VPS34 ubiquitination in CVDs is currently lacking, its regulators—such as UBC13 and NEDD4—are known to be involved in angiogenesis [47, 48], vascular remodeling [49] and hypertension [50], suggesting the potential role of VPS34 ubiquitination in CVD progression.

#### VPS34 Activation by SUMOylation

SUMOylation is the PTM of lysine residues with the ubiquitin-related small ubiquitin-like modifier (SUMO). First reported in 2014 [46], SUMOylation of VPS34 by KRAB-associated protein 1 on Lys840 (Fig. 1C, residues in green, Table 1) strengthened the interactions between VPS34 and other VPS34 complex subunits and thus enhanced the activity. Recent reports showed that SUMOylation of VPS34, with evidence of enhanced formation of VPS34 complex, increased in the vascular smooth muscle of mice with experimental pulmonary hypertension [21], indicating VPS34 activation through SUMOylation is associated with vascular smooth muscle cell hyper-proliferation in PAH.

####  VPS34 Activation through Complex Formation

In addition to VPS34 PTM, forming VPS34 complexes is essential for VPS34 activation. In mammalian cells, VPS34 can form two major heterotetrameric complexes [32], VPS34 Complex I and II (Fig. 3B, C). The core of both VPS34 complexes is VPS34/VPS15/Beclin 1, and they both facilitate the production of PtdIns3P. Complex I [formed by VPS34/VPS15/Beclin 1 and ATG14L] has been found to promote the autophagy pathway predominantly. In contrast, Complex II [formed by VPS34/VPS15/Beclin 1 and UV irradiation resistance-associated gene (UVRAG)] has been found to participate in various endocytic pathways [32, 51, 52].

Interestingly, co-expression of VPS34 and VPS15, even with a lower level of VPS34, leads to a 2.5-fold increase in VPS34 activity compared to the single transfection of VPS34 in HEK-294 T cells, indicating that VPS15 is a significant regulator of VPS34 activation [53]. In contrast, removing VPS15 by CRISPR reduced the expression of VPS34, ATG14, and Beclin 1 in HEK293A cells [54]. Similarly, cre-mediated mouse VPS15 excision in vivo also dramatically drops VPS34 and Beclin 1 protein levels in mouse embryonic fibroblasts (MEF) [55]. These data suggest that VPS15 regulates VPS34 expression, promotes the stability of VPS34 complexes, and is crucial in VPS34 activation. In addition to VPS15, Beclin 1 is essential in forming VPS34 complexes. Dissociation of Beclin 1 and VPS34 by protein phosphatase 6 inactivates VPS34 in MEF [56]. Selective pharmacological disruption on Beclin 1-ATG14L interaction, or the dissociation of VPS34 Complex I, by Compound 19 significantly decreased the accumulation of light chain 3 (LC3)-I and II, widely used autophagosome markers. However, Compound 19 did not disrupt Beclin 1-UVRAG interaction; thus, endocytosis regulated by VPS34 Complex II was unaffected [51]. Collectively, these data suggest that the Beclin 1-dependent formation and stability of a VPS34 complex is important for the VPS34 activation and function.

#### Sex Hormone-mediated VPS34 Regulation

Sex differences in disease onset have been observed across various CVDs, including hypertrophic cardiomyopathy [57], heart failure [58] and pulmonary hypertension [59–64]. Our research, alongside others, has highlighted sex-dependent pharmacological responses in treating pulmonary hypertension in both patients [65] and rodent models [66]. Nuclear sex hormone receptors, such as androgen receptors and estrogen receptors, are known to interact with PI3Ks, including VPS34 [67–69]. For instance, the antiandrogen enzalutamide promotes the interaction between Beclin 1 and the androgen receptor, leading to the inactivation of VPS34 complexes in prostate cancer C4-2 cells [70]. Moreover, human genome mapping (GRCh38/hg38) suggests that the androgen receptor regulates VPS34 transcription by binding to the VPS34 gene [71]. In support of this prediction, synthetic androgenic reagent 17α-Methyltestosterone downregulated VPS34 (*pik3c3*) mRNA levels in brains from female, but not male, *Gobiocypris. rarus *[72], further indicating interactions between androgen receptor and VPS34. Recent studies have shown a negative correlation between circulating testosterone, an endogenous androgen receptor ligand, and the severity of PAH in premenopausal female patients [59, 73], suggesting that VPS34 activation may potentially exhibit a sex hormone-dependent pattern in PAH. PAH research, for example, mostly focuses on the effects of estrogen and related receptors on disease progression, which, to our knowledge, does not interact with VPS34. Therefore, more rigorous research to understand the sex hormone-mediated VPS34 regulation in CVDs, including PAH, is needed and will shed light on future CVD therapy development.

### Functions of VPS34

VPS34 regulates three major cell signaling pathways: the VPS34/mTOR pathway, autophagy, and endocytosis. Briefly, VPS34 regulates mTOR activation through its upstream negative regulators, while mTOR can also regulate VPS34 Complex II through UVRAG phosphorylation. In addition, VPS34 activation promotes intracellular PtdIns3P production, a crucial step in the formation of autophagosomes and endosomes. PtdIns3P binds to proteins containing FYVE and PX domains, facilitating vesicle trafficking and protein sorting [13, 32, 74, 75]. The inhibition of VPS34 compromises the co-localization of FYVE/PX and endosomes by affecting PtdIns3P production [76], leading to the inhibition in autophagy and endocytosis. Here, we summarized the function of VPS34 in the three major cell signaling pathways and their roles in cardiovascular diseases.

#### Interactions between VPS34 and mTOR

**Fig. 4:**
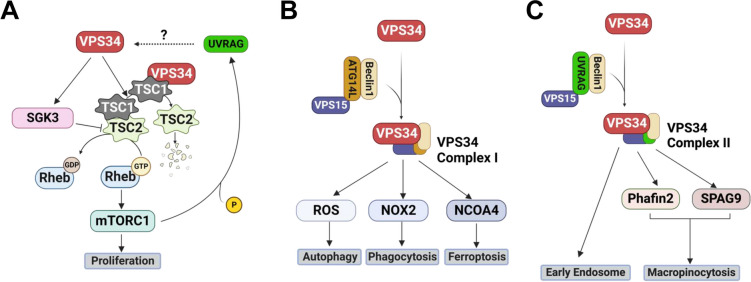
Schematic VPS34 pathway. **A** VPS34 regulates the mTOR pathway by affecting the TSC1/TSC2 complex. The activated mTORC1 can phosphorylate UVRAG, which, together with VPS15 and Beclin1, is an important component of VPS34 Complex II. **B** VPS34 Complex I, composed of VPS34 and VPS15/Beclin1/ATG-14L, regulates the autophagy pathway. **C** The VPS34 Complex II mainly promotes the endocytosis pathway, which can also regulate cell proliferation through a TSC2/mTOR-independent pathway

Activation of mTOR is known to be responsible for increased cell proliferation [7, 77, 78]. The tuberous sclerosis complex (TSC) dimer, composed of TSC1 and TSC2, is the critical upstream negative regulator of mTOR [79, 80]. The activation of TSC1/TSC2 stimulates the transition of Ras homolog enriched in brain (RheB)-GTP to RheB-GDP, thus inactivating mTOR complex 1 (mTORC1) [81]. VPS34 promotes mTOR activation through binding to TSC1 and disrupting TSC1/2 heterodimerization, which leads to TSC2 degradation and, consequently, mTOR activation in NIH3T3 fibroblast cells [9] (Fig. 4A). The His868Arg mutation of VPS34 has been shown to increase VPS34 activity independent of VPS34 complex formation significantly [9]. The transfection of VPS34-His868Arg into NIH3T3 cells led to increased VPS34-TSC1 binding, TSC1/2 heterodimer dissociation and consequent degradation of TSC2, formation of RheB-GTP, and mTOR activation [9]. Moreover, activation of VPS34 further activates SGK3 (serum/glucocorticoid regulated kinase 3), which phosphorylates and, thus, inhibits TSC2 from activating mTORC1 directly [82]. However, whether the VPS34 complex or VPS34 alone is required for the VPS34-TSC1 binding needs further investigation.

mTORC1 can also directly regulate VPS34. For example, mTORC1-mediated Ser550 and Ser571 phosphorylation of UVRAG, an essential component of VPS34 Complex II, activated VPS34 and enhanced cell survival after long-term starvation [83]; however, mTORC1-induced Ser498 phosphorylation of UVRAG inactivated VPS34 and decreased endosome maturation, which promoted the proliferation of HCT116, a colon cancer cell line [84]. The above interactions indicated a complicated regulation loop between mTOR and VPS34.

Importantly, the crosstalk among Class I PI3K/Akt/mTOR and VPS34/mTOR pathway activation, associated with increased cell proliferation and pulmonary arterial remodeling in PAH, has previously been reported by us and others [7, 8, 14, 79, 85] and the mTOR inhibitor, sirolimus, is in clinical trials to treat pulmonary hypertension with positive results [86]. These data suggested that mTOR and potentially VPS34 play important roles in cardiovascular cells; however, more research is needed to reveal if mTOR/VPS34 activation also contributes to the hyper-proliferation of pulmonary vascular cells in PAH.

#### VPS34 Complex I Regulates Autophagy

Autophagy is a crucial cell catabolic process that dictates cytoplasmic material degradation [87, 88]. VPS34 Complex I, recruited to the phagophores, is a known upstream regulator of autophagy [52] (Fig. 4B). Canonical autophagosome formation includes five main steps: initiation, nucleation, elongation, fusion, and degradation. The VPS34 Complex I produces PtdIns3P, which then participates in autolysosome nucleation with several downstream regulators [89]. Deleting VPS34 in MEF cells led to autophagy flux block and decreased autophagic degradation, resulting in compensatory accumulation of autophagy markers LC3-II and p62 [23]. Meanwhile, the activation of VPS34 by SLAMF1 (signaling lymphocytic activation molecule family member 1) in myeloid cells increased PtdIns3P, stimulated NADPH oxidase 2 (NOX2), which plays an important role in neutrophil functions and the LC3-associated phagocytosis, and triggered reactive oxygen species (ROS) accumulation, crucial signal transducers in autophagy [89–91]. Recently, ferroptosis, a special form of autophagy, has gained broad interest. One of the regulators of ferroptosis is nuclear receptor coactivator 4 (NCOA4) [92]. Selective pharmacological inhibition of VPS34 in DLD1 cells (a human colon cancer cell line) decreased NCOA4-mediated ferritin degradation, indicating NCOA4-dependent ferroptosis is regulated by VPS34 [93]. Recent research showed doxorubicin induces cardiotoxicity by promoting ferroptosis in cardiomyocytes [94–97], which raised the question of whether VPS34 inhibition could reduce cardiotoxicity induced by doxorubicin-associated ferroptosis. There are indications that, at least in the end stage of PAH, left ventricle mass loss is associated with upregulated autophagy [98]. In addition, autophagy is also increased in experimental pulmonary hypertensive animals [99, 100]. On the other hand, reports also showed the protective role of autophagy in pulmonary hypertension. For instance, it is shown that osteopontin-induced inhibition of autophagy promotes PI3K-Akt-mediated PAVSMC proliferation in hypoxic pulmonary hypertension [101]. It is hypothesized that the degree of autophagy contributes to the controversial effects in PAH and other cardiovascular diseases [102]. Further investigation to understand the function of VPS34-mediated autophagy will enrich our understanding of CVD progression and could help improve CVD treatments.

#### VPS34 Complex II Regulates Endocytosis

The endocytic pathway controls many downstream signaling cascades with different functions, including membrane trafficking and deformations [103]. VPS34 Complex II recruited by early endosomes regulates endocytosis, specifically synaptic vesicle cycling [104] and apical trafficking [105], in which PtdIns3P produced by VPS34 Complex II plays an important role in forming early endosomes [106] (Fig. 4C). VPS34 deletion in mouse proximal tubule cells almost completely prevented the formation of basolateral plasma membrane infoldings, which is associated with decreased early and recycling endosomes, clathrin-coated vesicles, and decreased membrane proteins [107]. Macropinocytosis, the endocytosis process that facilitates nonspecific bulk extracellular fluid internalization, is activated in cancer [108–110] and in TSC2-deficient cells [111]. VPS34-regulated PtdIns3P is essential in early macropinosome and endosome localization [112]. For example, the inhibition of PtdIns3P production by VPS34 inhibitor SAR405 prevented the second phase of Phafin2 localization and the downstream colocalization of Sperm Associated Antigen 9 (SPAG9), two vital proteins recruited in macropinosome formation and maturation, leading to the abolishment of Macropinocytosis [112, 113]. Additionally, although canonical cell proliferation can be regulated by VPS34 through downregulating TSC2 and activating mTORC1 (Fig. 4A), research also showed that VPS34 inhibition decreased micropinocytosis in TSC2-null cells and suppressed TSC2-deficient tumor growth in mice [111]. These results indicate the importance of TSC2/mTORC1-independent VPS34-mediated endocytosis in regulating cell proliferation.

Research has shown the important role that endocytosis plays in cardiovascular health and disease. For example, sorting nexin (SNX), an endocytic protein regulating protein sorting, maintains blood pressure by regulating dopamine receptors [114, 115]. Although the role of VPS34 in blood pressure maintenance through SNX remains unclear, evidence has shown that VPS34 positively regulates SNX17 expression and further regulates the recycling of endosomes [116]. Moreover, platelet-derived growth factor (PDGF)-BB, a stimulator of vascular smooth muscle cells (VSMC), proliferation, is found to be regulated by the endocytic regulator LRP1 (low density lipoprotein receptor-related protein 1), which is associated with abdominal aortic aneurysm (AAA) [117]. Interestingly, the overexpression of PDGF pathway genes is associated with upregulation of VPS34 in kidney cancer [118]; however, whether such association is also true in pulmonary vascular or aortic smooth muscle cells remains to be tested.

### Dysregulated VPS34 in Cardiovascular Cell Pathology

As we have summarized above, VPS34 has been reported to participate in the development or prevention of cardiovascular diseases. Here, we summarized three CVD-related conditions and their association with VPS34, vascular smooth muscle cell hyper-proliferation, cardiomyopathy, and thrombosis (Table 2).

**Table 2:** VPS34 in CVD

Pathology	Diseases	VPS34 regulation	Involved signaling	References
Vascular smooth muscle cell hyperproliferation	Abdominal aortic aneurysm; Pulmonary hypertension	Activation	Osteopontin↑, Angiotensin II↑, VPS34 SUMOylation	[21, 119, 120]
Cardiomyopathy	Ischemic cardiomyopathy	Activation	Hsp27) ↓ Gα_q_↑	[22–24, 121]
	Hypertrophic cardiomyopathy	Inactivation	CryAB↑ Autophagy↓	
MK cell maturation; Platelets over-activation of platelets	Thrombosis; Chronic thromboembolic pulmonary hypertension	Activation	NEDD?	[25, 45, 122]

#### VPS34 activation promotes vascular smooth muscle cell hyper-proliferation

VPS34 activation is associated with uncontrolled proliferation in cancer cells [123, 124], and research also indicates that it contributes to the hyper-proliferation of diseased VSMC, which is featured in CVDs, such as AAA and pulmonary hypertension.

Specifically, VPS34 gene expression was markedly increased in aorta wall tissues from patients with AAA, which was associated with increased mRNA and protein levels of osteopontin, known to regulate tumor cell proliferation [119]. Similarly, the overexpression of osteopontin in human VSMC from healthy donors induced VPS34 upregulation [120]. Additionally, angiotensin II (AngII)-induced vascular remodeling is associated with increased VPS34 expression and VPS34 activation in mouse aortas. Transmembrane member 16A (TMEM16A) ameliorated AngII-induced mouse aorta SMC hyper-proliferation, but the overexpression of VPS34 abolished the effect of TMEM16A. This indicates that abnormal VPS34 upregulation is responsible for VSMC hyperproliferation in mouse aorta [125]. Moreover, Yao et al. reported PAVSMC hyper-proliferation was associated with enhanced VPS34 SUMOylation, and therefore VPS34 activation, as the cells were exposed to hypoxia. They further showed that the hypoxia-induced VPS34 activation was enhanced by SUMO1 overexpression [21], indicating SUMOylation-induced VPS34 activation is responsible, at least in part, for PAVSMC hyper-proliferation in the development of pulmonary vascular remodeling and PAH. Preliminary studies from our research showed that knockdown of VPS34 by siRNA in PAVSMC from patients with PAH was associated with increased TSC2 and consequent mTOR inactivation, decreased accumulation of VPS15, and decreased proliferation and survival [20]. We hypothesize that VPS34 and its increased activation are responsible for the hyper-proliferation in the diseased PAVSMC from patients with PAH; however, the mechanisms by which the VPS34/mTOR axis is activated, especially the regulation of TSC2 by VPS34, in PAVSMC is yet to be unveiled.

#### Dual Role of VPS34 in Cardiomyopathy

The onset phase of heart failure is typically accompanied by cardiac hypertrophy, which is characterized by increased sizes of cardiomyocytes and the thickness of ventricular walls [126]. VPS34 plays a complicated role in regulating the proliferation and survival of cardiomyocytes depending on the type of cardiomyopathy present.

Conditional knock-out of *VPS34* in cardiomyocytes in mice (Mck/Vps34^−/−^) increased the heart size and heart/body ratio, which was associated with the accumulation of αB-crystallin (CryAB), left ventricular hypertrophy, and decreased heart contractility [23]. It was also found that myofibril continuity was compromised with defects in protein degradation in the hearts of Mck/Vps34^−/−^ mice. These alterations were further observed in patients with hypertrophic cardiomyopathy; in these patients, VPS34 protein level was decreased in 7 out of 18 heart samples, a significantly larger proportion than in healthy control samples [22]. The impaired autophagy induced by VPS34 deficiency in cardiomyocytes is hypothesized to contribute to CryAB/desmin aggregation and consequent myofibril disarray and pathological hypertrophy. However, in patients with ischemic cardiomyopathy, as opposed to hypertrophic cardiomyopathy, the VPS34 protein level was increased in 10 out of 15 heart samples [22]. Moreover, VPS34 expression and VPS34 complex activity were significantly increased in the hearts of mice with heat shock protein 27 (Hsp27) knock-out, which is associated with heart hypertrophy. Three weeks of pharmacological VPS34 inhibition was protective against heart hypertrophy induced by Hsp27 knock-out in mice [24]. Additionally, Gα_q_ is known to be involved in the induction of heart failure [127, 128]. Liu et al. reported that the activation of VPS34 was also found in transgenic mice with cardiomyocyte-specific expression of Gα_q_-Gln209Leu, a constitutively active mutation, which is associated with increased autophagy marker p62 expression, decreased cardiac contractility, and heart failure [121]. The opposing role that VPS34 plays in different types of cardiomyopathies indicates the complex role of VPS34 in cardiovascular health and CVDs.

This dual role of VPS34 in cardiomyocytes might rely on the different mechanisms of VPS34 complexes. As discussed above, the activation of VPS34 Complex I promotes the initiation of autophagy [129], while the VPS34 Complex II is found to interact with mTORC1 and promotes endocytosis [83, 130]. The mechanisms by which Complexes I and II interact with each other in cardiomyocytes are not clear; however, the balance between the VPS34-autophagy and the VPS34/mTOR axes should be considered for further research in mechanisms of heart failure and therapy development. It is worth noting here that the degree of autophagy may exhibit either protective or pathological effects in cardiovascular cells. Given the important role of VPS34 in autophagy regulation, the homeostasis of VPS34-activation-induced autophagy should also be carefully investigated.

#### VPS34 Induces Thrombosis

Platelets, produced from megakaryocytes (MKs), are the first responders of blood vessel injuries; however, over-activation of platelets leads to thrombosis, or blood clots, that limit blood flow in the circulation system [131, 132]. MK/platelet-specific deletion of VPS34 in mice, which was associated with decreased protein levels of VPS15 and Beclin 1, reduced arterial thrombosis, and impaired platelet aggregation [25]. Moreover, in VPS34-deficient mouse MKs, transferrin and fibrinogen endocytosis were significantly reduced, which was associated with increased granule release responses to acute platelet stimulation and decreased thrombus growth [26]. Importantly, the colocalization of VPS34 with CD61 and VPS34-derived PtdIns3P promoted the maturation of MKs [133]. These results strongly indicate that VPS34 is required for MK maturation and thrombus formation. VPS34 inhibition by 3-Methyladenine decreased the platelet adhesion induced by collagen and thrombin [134], showing that VPS34 inhibitors could be used to prevent thrombosis.

The lung is one of the major sites of MK-origin platelet release [135]. Deep vein thrombosis followed by pulmonary emboli leads to chronic thromboembolic pulmonary hypertension (CTEPH) [136]. Whether VPS34 plays a role in promoting thrombosis in CTEPH is not known. Recently, research showed that the upregulation of NEDD9 in pulmonary arterial endothelial cells facilitated the adhesion of platelet and endothelial cells in the pulmonary vasculature from CTEPH patients [137]. It remains unclear if the regulation or function of VPS34 is associated with NEDD9 in pulmonary vascular endothelial cells or MKs/platelets in human cells. One report showed that miR-125b inhibited the PI3K/Akt pathway via downregulating NEDD9 in Capan1 cells, a human pancreatic cell line [122], indicating a potential link between NEDD9 and PI3K regulation. Although the direct interaction between NEDD9 and VPS34 activation remains to be elucidated, interestingly, NEDD4, an E3 ubiquitin-protein ligase of the NEDD family, stabilizes VPS34 and promotes its activation [45]. We do not know if NEDD4 is involved in platelet-endothelial interaction; however, VPS34 regulation could be a future direction for mechanism study and therapy development of CTEPH.

## Current Pharmacological Approaches in Regulating VPS34

### VPS34 Inhibitors

In the past decade, the search for VPS34 pharmacological inhibitors has been of great interest for cancer research and therapy. This has led to several highly selective reagents, with low in vitro clearance and a high fraction unbound in plasma (Table 3).

**Table 3.** VPS34 inhibitors

Structure (Name)	VPS34 IC_50_	Pharmacology	References
	1.2 nM	SAR405 prevented autophagy and decreased cancer cell proliferation with rapamycin	[76]
	4 nM	VPS34-IN-1 repressed the stemness gene expression in liver cancer stem cells	[138, 139]
	2 nM	VPS34-IN-2 (Compound 32) significantly decreased the histological tumor marker, cytoplasmic granular staining, in tumor xenografted on mice	[140]
	19 nM	Autophinib inhibited autophagy and induced cell deaths in human breast cancer epithelial cells (MCF7)	[141]

SAR405, one of the first selective VPS34 inhibitors, interacts with the catalytic kinase domain and the ATP-binding pocket of VPS34, without modifying the confirmation [76]. SAR405 treatment significantly decreased fibroblast proliferation [142] and synergized osteosarcoma cell apoptosis induced by celecoxib [143]. These studies showed that VPS34 inhibitors could potentially benefit diseases with cell hyper-proliferation. Our preliminary data also indicates SAR405 decreased the hyper-proliferation and survival of PAVSMC from patients with PAH and reduced pulmonary vascular remodeling in experimental pulmonary hypertension in male mice [20], further indicating the potential clinical uses of VPS34 inhibitors. Additionally, our preliminary data suggested an Akt-dependent VPS34 activation in PAVSMC from patients with PAH [20], while Akt activation induced by hypoxia/Lipopolysaccharides (LPS) in dendritic cells was significantly reduced by 10°μM SAR405 [144]. These data, suggest a complex regulatory pathway between Akt and VPS34, whereby the regulation of VPS34 and Akt may form a feedforward loop in the disease state.

VPS34-IN-1, another VPS34 selective inhibitor, significantly decreased the expression of stemness genes in liver cancer stem cells (CSC) [138]. Moreover, VPS34-IN-1 treatment led to a decreased percentage of CD133 + (CSC surface marker) positive MHCC97H and Huh7 cells and resulted in smaller tumor sizes in a tumor xenograft model, indicating VPS34 inhibition attenuates cancer cell proliferation. Notably, the Class I PI3K inhibitor ZSTK474 increased liver CSC expansion via SGK3-mediated mTOR activation, a process that can be effectively blocked by VPS34-IN-1 [138]. Although CSCs are not derived from cardiovascular tissues, their hyperproliferation resembles the behavior of cells involved in vascular remodeling. Therefore, the potential synergistic effects of VPS34-IN-1 with ZSTK474, or other combinations, should be explored in hyperproliferative cardiovascular cells and relevant preclinical models.

Based on SAR405 and VPS34-IN-1, other potent, selective, and bioavailable VPS34 inhibitors have been developed by targeting residues Met682 and Phe612, both of which are located in the VPS34 kinase domain. This results in inhibitors that have a much lower selectivity to Class I PI3K α, β, δ, and γ [33, 145]. However, no VPS34-targeted therapies have yet advanced to clinical trials. Additionally, the current preclinical applications of VPS34 inhibitors remain largely confined to cancer research. This limitation is likely due to the previously limited availability of VPS34-specific inhibitors, while the pharmacological profiles of many newly developed compounds are still under investigation. Given VPS34's functions and regulation in cardiovascular health and disease we discussed above, it is essential to explore the therapeutic potential of selective VPS34 inhibitors in the context of CVDs.

### Potential of using VPS34 Inhibitors in Pulmonary Vascular Diseases

Although little is known about the role of VPS34 in pulmonary vascular diseases, data support VPS34's involvement in regulating PAVSMC proliferation in PAH and thrombosis formation in CTEPH. We and others have shown that mTOR activation drives increased proliferation of PAVSMC in PAH [8, 14, 146]. Current clinical studies to use mTOR inhibitors to treat PAH have shown promising results: (1) Phase I/II treatment of ABI-009 (sirolimus), loaded on albumin-bound nanoparticles, to five out of six patients with severe PAH showed good tolerance, and three out of five patients completed the trial showed amelioration of PAH [86, 147]; (2) a pilot clinical study of everolimus (42-O-(2-hydroxyethyl)-rapamycin) also showed it was tolerable in eight out of nine idiopathic PAH and CTEPH patients, and seven of eight patients exhibited improved pulmonary vascular resistance while six improved in the 6-min walk distance test [148]. However, rapamycin and its derivatives may lead to increased serum cholesterol, hyperglycemia, hyperlipidemia, insulin resistance, and type 2 diabetes [148], therefore, it is of great interest to look for reagents that could potentially decrease the dose of rapamycin while providing similar or synergic effects against pulmonary vascular pathologies. VPS34 could be a good target because of its role in mTOR regulation as discussed above. Ronan et al. showed that treatment of SAR405 significantly inhibited autophagy induced by the mTOR inhibitor AZD8055 and further decreased hyper-proliferation in cancer cells. Similarly, combination treatment of cancer cells with SAR405 and everolimus synergistically prohibited cell proliferation [76]. Given the similar cell signaling in cell hyper-proliferation in pulmonary vascular remodeling, it is also of great interest to investigate the effects of the combination therapy with mTOR and VPS34 inhibitors.

## Summary and Future Perspectives

VPS34 and its complexes regulate cardiovascular health and disease, influencing processes such as VSMC proliferation, cardiomyocyte survival, MK maturation, and platelet-endothelial interactions. Dysregulation of these processes is hypothesized to be associated with factors such as osteopontin-mediated VPS34 dysfunction, VPS34 SUMOylation, and imbalances in VPS34 complex formation. However, gaps remain in our understanding, particularly regarding the regulatory mechanisms of VPS34. Specifically, a significant area for future exploration is the interaction between VPS34 and the mTOR pathway, a key regulator of cellular metabolism and growth in cardiovascular cells. The relationship between VPS34 activation and downstream signaling through mTOR in cardiovascular cells could provide valuable insights into disease mechanisms, especially its role in regulating VSMC hyper-proliferation in vascular remodeling and cardiomyopathy. Furthermore, a deeper understanding of the interplay between VPS34-induced autophagy and mTOR activation may illuminate its role in cardiomyopathy progression across different pathological conditions. Dysregulated VPS34 has been shown to promote abdominal aortic aneurysm, pulmonary hypertension, cardiomyopathy, and thrombosis, which are hypothesized to be through VPS34-mTOR interaction and VPS34-regulated autophagy. Lastly, the role and regulation of VPS34 in CTEPH remain poorly understood. Given the established role of VPS34 in MK maturation and thrombosis, identifying upstream and downstream regulators of VPS34 may provide novel therapeutic opportunities for managing CTEPH.

Beyond these direct connections to CVD, other avenues of investigation into the role of of VPS34 could lead to improved CVD treatments. Our research and others have shown sex differences in the progression of many CVDs and responses to pharmacological treatment. As such, the regulation of VPS34 expression by androgen receptors should be investigated in sex-specific CVD research, given the potential of hormone-mediated VPS34 activation. Whereas impressive advancements in Class I PI3K or mTOR inhibitors have been made in recent years, combining VPS34 inhibitors as a combination therapy could be a promising approach to uncover further how VSMCs become hyperproliferative during disease progression. Such approaches may also offer new therapeutic strategies for treating CVD, particularly those involving unchecked vascular cell growth.

Statement of the Clinical Relevance

Cardiovascular diseases (CVDs) remain one of the foremost causes of morbidity and mortality worldwide, underscoring the urgent need for novel therapeutic interventions to address their complex underlying mechanisms. In this review, we focus on the regulation and function of VPS34/PIK3C3 in the context of CVD progression, including conditions such as pulmonary hypertension, cardiomyocyte survival, and thrombosis.

By understanding the molecular roles of VPS34 in various CVD-related pathways, there is potential to uncover novel therapeutic targets. For instance, our review highlights that selective inhibition of VPS34 may provide a new approach to mitigate vascular smooth muscle cell proliferation, potentially enhancing the efficacy of treatments already under clinical investigation. This, and other discussed advances in VPS34 and CVDs, could significantly impact therapeutic strategies aimed at controlling vascular remodeling and thrombosis. Additionally, further insights into VPS34's contribution to cardiomyopathies could pave the way for more effective management of heart disease.

Ultimately, this review consolidates the current body of knowledge and recent advancements surrounding VPS34's role in cardiovascular health, offering a unified perspective on its therapeutic potential in CVD treatment strategies.

## Data Availability

Not applicable.

## Abbreviations

AAA: Abdominal aortic aneurysm
AMPK: 5' AMP-activated protein kinase
AngII: Angiotensin II
CDK1: Cyclin-dependent kinase 1
CryAB: αB-crystallin
CSC: Cancer stem cells
CTEPH: Chronic thromboembolic pulmonary hypertension
CVD: Cardiovascular disease
Hsp27: Heat shock protein 27
LC3: Light chain 3 protein
LPS: Lipopolysaccharides
LRP1: Low density lipoprotein receptor-related protein 1
MEF: Mouse embryonic fibroblasts
MK: Megakaryocyte
mTOR: Mammalian target of rapamycin
mTORC: Mammalian target of rapamycin complex
NCOA4: Nuclear receptor coactivator 4
NEDD: Neural precursor cell expressed developmentally down-regulated protein
NOX2: NADPH oxidase 2
PAH: Pulmonary arterial hypertension
PAVSMC: Pulmonary arterial vascular smooth muscle cells
PDGF: Platelet-derived growth factor
PI3K: Phosphatidylinositol-3 kinase
PtdIns: Phosphatidylinositol
PtdIns3P: Phosphatidylinositol 3-phosphate
PTM: Posttranslational modification
RheB: Ras homolog enriched in brain
ROS: Reactive oxygen species
SGK3: Serum/glucocorticoid regulated kinase 3
SLAMF: Signaling lymphocytic activation molecule family member
SNX: Sorting nexin
SPAG9: Sperm associated antigen 9
SUMO: Small ubiquitin-like modifier
TMEM16A: Transmembrane member 16A
TSC: Tuberous sclerosis complex
UVRAG: UV irradiation resistance-associated gene
VPS34: Vacuolar Protein Sorting 34
VSMC: Vascular smooth muscle cells
